# GLI1 reduces drug sensitivity by regulating cell cycle through PI3K/AKT/GSK3/CDK pathway in acute myeloid leukemia

**DOI:** 10.1038/s41419-021-03504-2

**Published:** 2021-03-03

**Authors:** Cheng Zhou, Juan Du, Liang Zhao, Wei Liu, Tianming Zhao, Hui Liang, Peng Fang, Kaixuan Zhang, Hui Zeng

**Affiliations:** 1 Department of Hematology, Xiangya Hospital, Central South University, Changsha, Hunan 410008 China; 2 Department of Hematology, The First Affiliated Hospital of Jinan University, Guangzhou, Guangdong 510630 China

**Keywords:** Apoptosis, Acute myeloid leukaemia

## Abstract

Acute myeloid leukemia (AML) is a hematological malignancy with high incidence and recurrence rates. Gene expression profiling has revealed that transcriptional overexpression of glioma‐associated oncogene 1 (GLI1), a vital gene in the Hedgehog (Hh) signaling pathway, occurs in poor-prognosis AML, and high levels of phosphoinositide-3-kinase, regulatory subunit 1 (PIK3R1) and AKT3 predict shorter overall survival in AML patients. In this study, we discovered that GLI1 overexpression promotes cell proliferation and reduces chemotherapy sensitivity in AML cells while knocking down GLI1 has the opposite effect. Moreover, GLI1 promoted cell cycle progression and led to elevated protein levels of cyclins and cyclin-dependent kinases (CDKs) in AML cells. By luciferase assays and co-immunoprecipitation, we demonstrated that the PI3K/AKT pathway is directly activated by GLI1. GLI1 overexpression significantly accelerates tumor growth and upregulated p-AKT, CDK4, and cyclinD3 in vivo. Notably, the GLI1 inhibitor GANT61 and the CDK4/6 inhibitor PD 0332991 had synergistic effects in promoting Ara-c sensitivity in AML cell lines and patient samples. Collectively, our data demonstrate that GLI1 reduces drug sensitivity by regulating cell cycle through the PI3K/AKT/GSK3/CDK pathway, providing a new perspective for involving GLI1 and CDK4/6 inhibitors in relapsed/refractory (RR) patient treatment.

## Introduction

Acute myeloid leukemia (AML) is a hematological malignancy with poor overall clinical outcome. Chemotherapy, such as Ara-c combination chemotherapy, is the most commonly used treatment for this disease. The high recurrence rate of AML is largely due to incomplete eradication of leukemia stem cells (LSCs) by conventional chemotherapy. LSCs, which give rise to leukemic blasts, are resistant to chemotherapy and responsible for disease relapse^1^. New targets and therapeutic approaches are needed. Among them, targeted therapy has proven to be a promising research area for AML treatment^2–4^.

Dysregulated signaling pathways are closely related to AML resistance and relapse. Accordingly, a number of lines of evidence have demonstrated that abnormal activation of the PI3K/AKT, Hedgehog (Hh), mTORC1, ERK/MAPK, STAT3/5, Wnt/β-catenin, and NF-κB pathways influence drug resistance in AML^5,6^. The Hh signaling pathway, which functions via two cellular receptors, patched (Ptch) and smoothened (Smo), is activated during embryogenesis and silenced in most adult tissues. Hh binds to Ptch, while Smo transduces the Hh signal, which is mediated by glioma-associated oncogene homolog (Gli) family members (GLI1, 2, 3)^7,8^. Among these family members, GLI1 acts as both a transcriptional activator and a Hh target gene^9^. Importantly, GLI1 is regarded as the most reliable indicator for Hh pathway activation^10^.

Dysregulation of Hh signaling has been implicated in a variety of cancers, including human pancreatic carcinoma, colorectal cancer, prostate cancer, small-cell lung cancer, basal cell carcinoma, and hematological malignancies^11^. Hh signaling contributes to tumor maintenance, growth, and resistance to chemotherapy in hematopoietic neoplasms, including diffuse large B-cell lymphoma and myeloid leukemia^12,13^. For example, GLI1 expression was positively correlated with the percentage of CD34^+^ cells in AML. Moreover, the GLI antagonist GANT61 reduced proliferative and colony-forming characteristics and displayed synergistic cytotoxicity with Ara-c in AML cell lines^9^. In addition, the Hh pathway is important for the survival and expansion of Bcr-Abl^+^ LSCs^14^. GLI1 was reported to be over-expressed in myeloid dysplastic disease (MDS) during disease progression^15^ and was an independent predictor of poor outcome in chronic lymphocytic leukemia^16^. In a mouse model, activation of Hh signaling led to accelerated progression and leukemic transformation of FLT3-ITD-driven indolent myeloproliferative disease^17^. As a result, Hh inhibitors have been explored and shown to be effective in preclinical studies for MDS and AML, and preliminary data from ongoing clinical trials using SMO inhibitors demonstrate promising antitumor activity^18,19^. In 2018, glasdegib, an SMO inhibitor, was approved by the FDA for the treatment of AML and MDS.

The phosphoinositide 3‑kinase (PI3K)/protein kinase B (AKT)/rapamycin kinase (mTOR) signaling pathway is involved in cell growth, proliferation, and differentiation and influences the occurrence, development, treatment, and outcome of various cancers. Numerous studies have reported that the PI3K/AKT/mTOR signaling pathway is constitutively activated in leukemia cells^20,21^, and hyperactivation of the PI3K/AKT pathway has been associated with drug resistance and relapse in AML cells^5^. The disease-free survival and overall survival rate of AML patients are significantly reduced in those with an up-regulated PI3K/AKT signaling pathway^22,23^. Thus, the PI3K/AKT/mTOR signaling pathway has become a new potential target for tumor therapy^24,25^. Selective small-molecule inhibitors of PI3K/AKT/mTOR signaling are now being intensively tested in clinical trials of various tumors. Among them, PI3K inhibitors idelalisib and copanlisib have also been approved for the treatment of leukemia and lymphoma.

Crosstalk between the Hh signaling and PI3K/AKT pathways has been described in many cancers including melanoma, prostate cancer, non-melanoma skin cancer, glioma, and leukemia. For instance, the crosstalk between Hh/GLI and PI3K/AKT has an impact on GLI1 and GLI2 expression, protein stability, nuclear localization, and transcriptional activity^26–28^. Another study suggested that the Gli1/PI3K/AKT/NF-κB pathway plays a key role in resistance to radiation for refractory AML cells, and that inhibition of the Hh pathway sensitizes cells to radiation by overcoming radio-resistance^29^. We previously reported that the Hh and PI3K/AKT pathways are up-regulated in the AML RR population. Overexpression of GLI1 in AML cells led to increased AKT phosphorylation, which was attenuated by GLI1 inhibition. Moreover, GLI1 inhibition is sufficient to enhance AML drug sensitivity^30^.

In this study, we demonstrate that GLI1 accelerates mitosis by activating the PI3K/AKT/GSK3/CDK pathway, resulting in hyperproliferation and drug resistance in AML cells. Additionally, we demonstrate that co-treatment with GANT61 and PD 0332991 promotes Ara-c sensitivity, which provides a potential treatment for AML-RR patients.

## Results

### GLI1 regulates AML cell proliferation and drug sensitivity

**Fig. 1:**
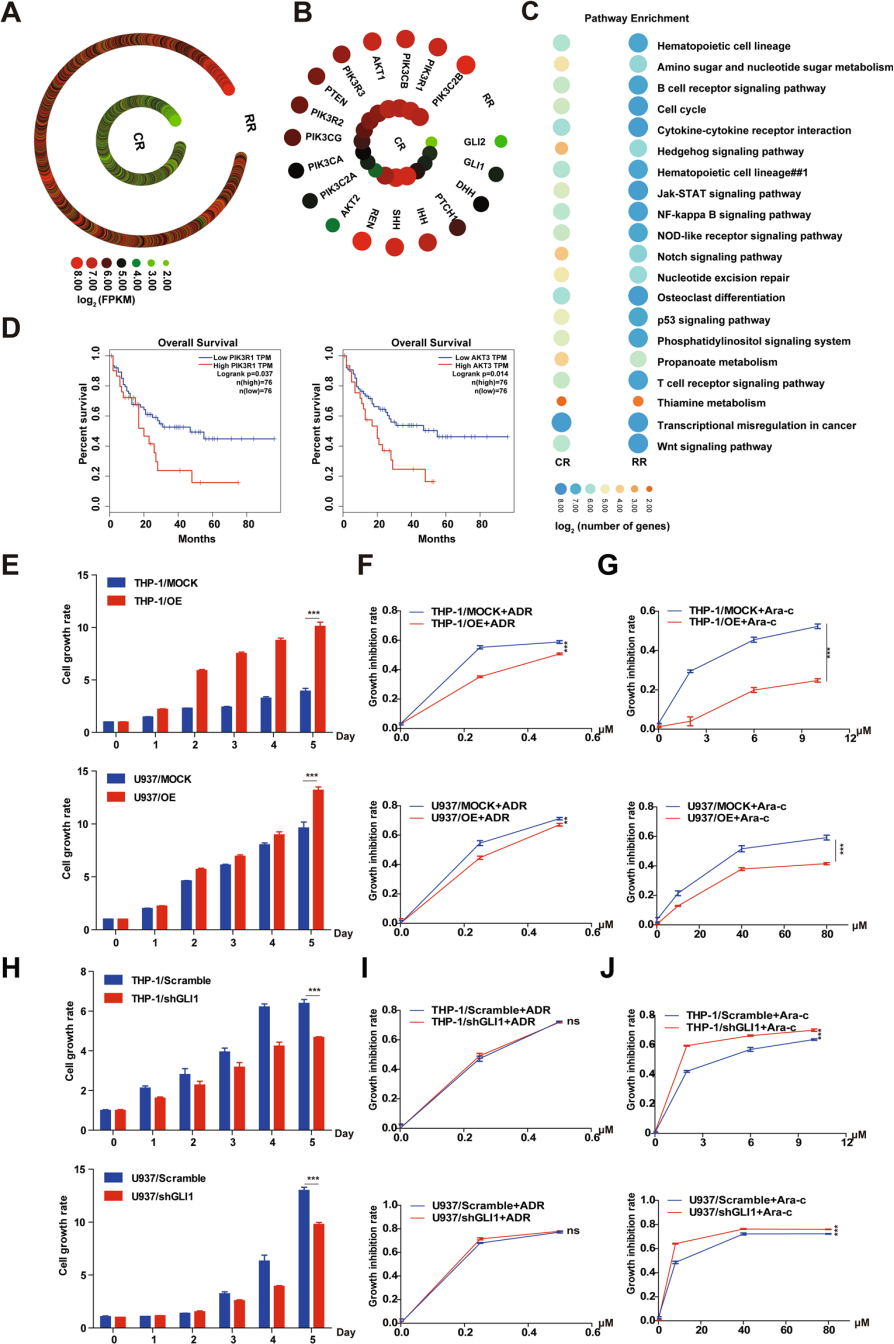
GLI1 regulates AML cell proliferation and reduces drug sensitivity. **A**, **B** Heatmap of all genes. Hh and PI3K/AKT pathway-related gene expression for 22 AML-RR patients and 22 AML-CR patients. **C** Pathways enriched in AML-RR compared to AML-CR patients with the top 20 pathways shown. **D** Kaplan–Meier curves of PIK3R1 and AKT3 for AML patients derived from the GEPIA database. **E** The growth rate of THP-1/MOCK/OE and U937/MOCK/OE cells was determined by Cell Counting Kit-8 assay. **F**, **G** Growth inhibition rates of THP-1 and U937 MOCK/OE cells treated with different concentrations of ADR/Ara-c. **H** The growth rates of THP-1/Scramble/shGLI1 and U937/Scramble/shGLI1 cells were determined by Cell Counting Kit-8 assay. **I**, **J** Growth inhibition rates of THP-1 and U937 Scramble/shGLI1 cells treated with different concentrations of ADR/Ara-c. p values were obtained by two-way ANOVA. *P < 0.05, **P < 0.01, ***P < 0.001, ns not significant.

To investigate whether the Hh and PI3K/AKT signaling pathways are dysregulated in AML-RR patients, we performed RNA-seq using pooled bone marrow samples collected from AML-RR patients (Fig. 1A). We found that signature gene of the Hh signaling pathway e.g., GLI1, PTCH1, SMO, and the PI3K/AKT pathway e.g., phosphoinositide-3-kinase, regulatory subunit 1 (PIK3R1), AKT1, AKT2, were upregulated in AML-RR compared to AML-CR patients, indicating that both the Hh and PI3K/AKT signaling pathways were up-regulated in AML-RR patients (Fig. 1B, C). By analysis of the clinical influence of PIK3R1 and AKT on AML with GEPIA, we discovered that the expression levels of PIK3R1 (also named PI3Kp85) and AKT3 were significantly higher in AML patients (*N* = 173) than in healthy people (*N* = 70) (Fig. S1A). Survival analysis demonstrated that AML patients with a high level of expression of GLI1, PIK3R1, and AKT3 had shorter overall survival (Kaplan–Meier survival analysis) (Figs. 1D and S1B).

Whether there is crosstalk between the Hh and PI3K/AKT pathways and if GLI1 plays a role in the interaction between the Hh and PI3K/AKT pathways in AML patients remains unknown. To explore the role of GLI1 in the interaction between Hh and the PI3K/AKT pathway in AML cells, we generated both GLI1 OE and KD cell lines from THP-1 and U937 cells by lentiviral transduction and confirmed the changes in GLI1 expression level by qPCR (Fig. S1C–E). Both overexpression and knockdown of GLI1 were further validated by Western blot at the protein level (Fig. S1F, G). First, to investigate the effects of GLI1 overexpression in AML cells, we assessed the cell growth rate of GLI1-overexpressing AML cells by CCK8 assay. GLI1 overexpression significantly increased the cell growth rate compared with that of control cells (Fig. 1E). Considering the Hh signaling pathway is related to drug resistance, we further examined the effects of elevated expression of GLI1 on the sensitivity of chemotherapy drugs in AML cell lines. Adriamycin (ADR) and Cytarabine (Ara-c) are the most frequently used chemotherapy drugs in AML treatment. Therefore, we examined whether upregulation of GLI1 reduces the sensitivity of AML cells to ADR and/or Ara-c. After treatment with ADR or Ara-c for 48 h, the growth inhibition rates were significantly lower in THP-1/OE and U937/OE cells compared with control cells (Fig. 1F, G).

Conversely, to investigate how GLI1 knockdown affects the growth rate of AML cells, we examined the growth of the GLI1 knockdown cell lines THP-1/shGLI1 and U937/shGLI1. GLI1 knockdown significantly repressed the growth rate of both cell lines compared with control cells (Fig. 1H). Subsequently, we also tested whether knockdown of GLI1 increases the sensitivity of AML cells to ADR and/or Ara-c. Surprisingly, no difference in the growth inhibition rate was observed for the GLI1 knockdown cell lines THP-1/shGLI1 and U937/shGLI1 when ADR was added (Fig. 1I). In contrast, the growth inhibition rate was significantly higher for THP-1/shGLI1 and U937/shGLI1 cells compared with control cells when cells were treated with Ara-c (Fig. 1J). Collectively, these data suggest that GLI1 plays an important role in regulating AML cell proliferation and sensitivity to chemotherapeutic drugs.

### Overexpression GLI1 promotes cell cycle progression by up-regulating cyclins, cyclin-dependent kinases (CDKs), and the PI3K/AKT pathway

**Fig. 2:**
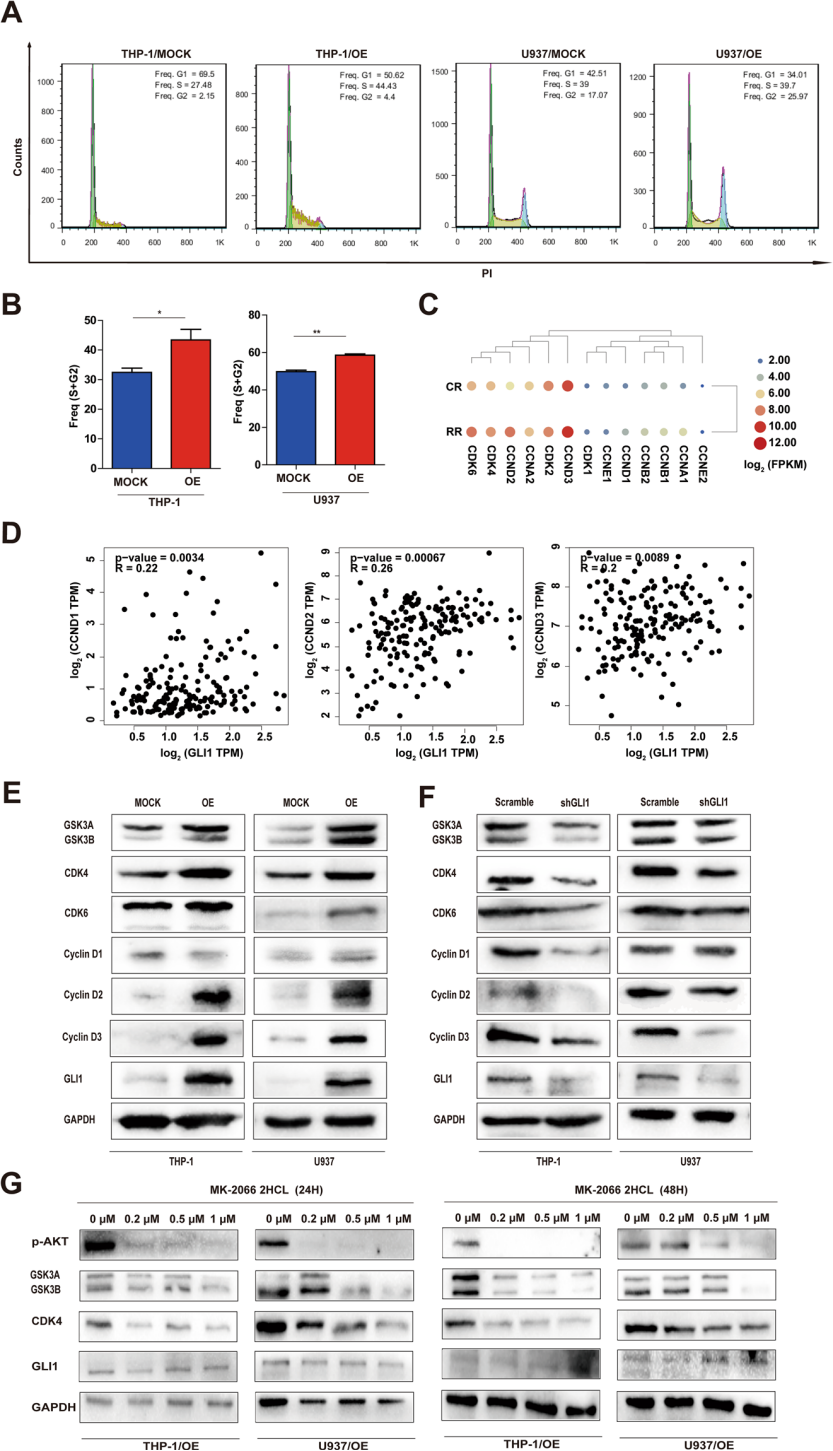
Overexpression GLI1 up-regulates cyclins and cyclin-dependent kinases. **A**, **B** Representative histograms (**A**) and quantification (**B**) of cell cycle phases of THP-1 and U937 OE/MOCK cells by FACS analysis. **C** Differentially expressed cell cycle-related genes in AML-RR and AML-CR patients from analysis of RNA-seq data. **D** The correlation between GLI1 and cyclin Ds in AML patients was evaluated using a non-log scale for calculation and a log-scale axis for visualization. The coefficient of correlation (*r*) and the *p*-value is indicated. **E**, **F** Protein expression of GSK3A/B (51/47 kDa), cyclin D1/D2/D3 (36 kDa/32 kDa/33 kDa), CDK4 (34 kDa), and CDK6 (36 kDa) in THP-1 and U937 MOCK/OE cells (**E**) and THP-1 and U937 Scramble/shGLI1 cells (**F**) by Western blot. **G** THP1/OE and U937/OE cells were treated with different concentrations of MK-2206 2HCL (0, 0.2, 0.5, and 1 μM) for 24 h (left) and 48 h (right). Cell lysates were analyzed by Western blotting for the p-AKT (65 kDa), GSK3A/B, and CDK4 protein levels. *P* values were obtained by two-way ANOVA. *P < 0.05, **P < 0.01, ***P < 0.001, *ns* not significant.

Ara-c is a deoxynucleoside analog that is incorporated into replicating DNA during the S phase of the cell cycle^31^. To test whether overexpression of GLI1 promotes cell cycle progression in AML cells, we assessed the cell cycle status of AML cell lines overexpressing GLI by BrdU uptake (Fig. 2A). We found that GLI1 overexpression significantly increases the percentage of AML cells in S + G2 phase (Fig. 2B), indicating overexpression of GLI1 accelerates cell cycle progression. To determine how GLI1 accelerates the cell cycle, we analyzed transcriptomic data from AML-RR patients. We found that cyclins and CDKs were up-regulated in AML-RR patients, particularly cyclin Ds and CDKs (Fig. 2C). By using GEPIA to analyze the correlation between GLI1 and cyclin Ds, CDKs, and GSK3α/β, we found that the cyclin D, CDK6, and GSK3α/β levels were positively correlated with GLI1 in AML patients (Figs. 2D and S2A). Therefore, overexpression of GLI1 may accelerate the cell cycle by upregulating the cell cycle regulators GSK3α/β, Cyclin Ds, and CDKs. To test whether GLI1 regulates the expression of cell cycle regulating proteins, we examined the expression levels of GSK3α/β, Cyclin Ds (cyclin D1, D2, and D3), CDK4, and CDK6 in THP-1/OE, U937/OE, THP-1/shGLI1, and U937/shGLI1 cells by Western blot and qPCR. Our results demonstrated that GLI1 overexpression significantly elevated the protein levels of GSK3α/β, Cyclin D (cyclin D2 and D3), CDK4, and CDK6 compared with control cells (Figs. 2E and S2B). Conversely, knocking down GLI1 decreased the protein levels of GSK3α/β, Cyclin Ds (cyclin D2 and D3), CDK4, and CDK6 compared with control cells (Figs. 2F and S2C). Furthermore, protein-protein interactions predicted by the STRING database demonstrated that PI3K/AKT could unidirectionally regulate the expression of cell cycle-related proteins after passing through the GSK3β gene knot (Fig. S3A). Thus, we proposed that inhibition of AKT would reverse the upregulation of cell cycle regulators induced by GLI1 overexpression without affecting GLI1. To test this hypothesis, THP1/OE and U937/OE cells were treated with different concentrations of MK-2206 2HCL, an AKT inhibitor. As expected, MK-2206 2HCL could reverse the increase in GSK3α/β and CDK4 resulting from GLI1 overexpression but spared the GLI1 expression level (Fig. 2G). By GEPIA analysis, we also found that cyclin Ds, CDKs, and GSK3α/β levels were positively correlated with AKT in AML patients (Fig. S3B–D). Collectively, our results suggest that overexpression of GLI1 promotes cell cycle progression by upregulating cell cycle regulators and the PI3K/AKT pathway.

### GLI1 increases phosphorylation of essential components of the PI3K/AKT pathway and directly binds PIK3R1

**Fig. 3:**
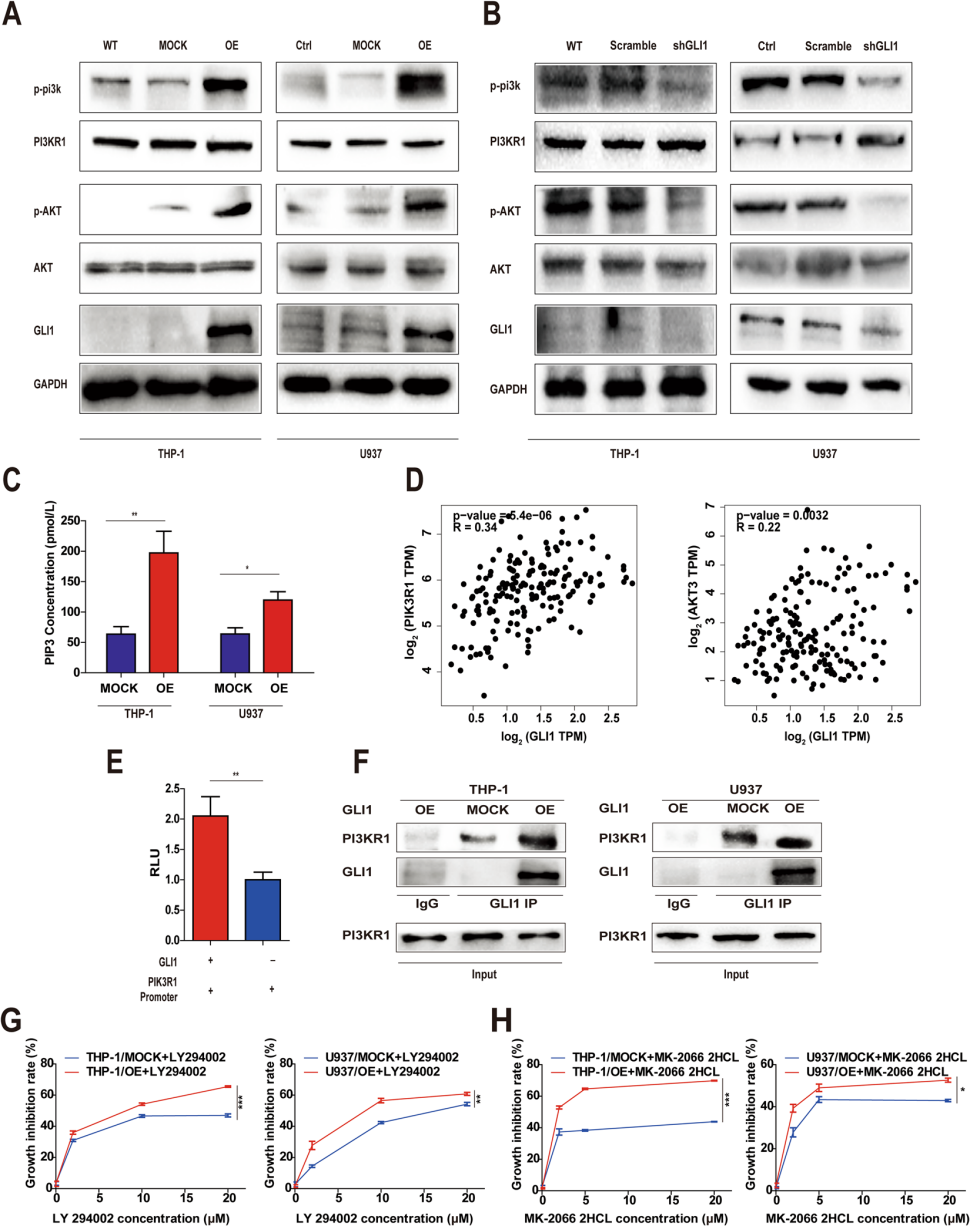
GLI1 activates the PIK3R1 reporter and promotes GLI1-PIK3R1 binding. **A**, **B** The phosphorylation levels of PI3K and AKT in THP-1 and U937 cells with stable GLI1 overexpression and stable GLI1 knockdown. GAPDH was used as a loading control. WT, wild-type cells without treatment. **C** PIP3 levels in THP-1 and U937 MOCK/OE cells. **D** Correlations between GLI1 and PIK3R1 and GLI1 and AKT3 in AML patients were evaluated using a non-log scale for calculation and a log-scale for visualization. **E** The ability of PIK3R1 to activate the GLI1 reporter was assessed in 293 T cells. As a control, transcriptionally inactive GLI1 was tested. **F** Western blot analysis of the PI3KR1 and GLI1 protein expression levels in THP-1 and U937 MOCK/OE cells (Co-IP). **G, H** The viability of THP-1 and U937 MOCK/OE cells treated with different concentrations of LY249002 or MK-2206 2HCL was determined by CCK8. *P* values were calculated by the two-tailed Student’s *t*-test and two-way ANOVA. *P < 0.05, **P < 0.01, ***P < 0.001, ns not significant.

Because we found that GLI1 may regulate the cell cycle through the PI3K/AKT pathway, we further sought to validated whether GLI1 regulates the phosphorylation of essential components of the PI3K/AKT pathway by Western blotting. Interestingly, stable overexpression of GLI1 increased the phosphorylation of PI3K and AKT in THP-1/OE and U937/OE cells but did not alter their expression level (Fig. 3A). Conversely, stable knockdown of GLI1 attenuated the phosphorylation of PI3K and AKT in THP-1/shGLI1 and U937/shGLI1 cells (Fig. 3B).

We next sought to explore the molecular mechanism underlying how GLI1 affects the PI3K/AKT signaling pathway. We found that GLI1 overexpression resulted in increased phosphatidylinositol (3,4,5)-trisphosphate (PIP3) levels in THP-1/OE and U937/OE cells (Fig. 3C), and the expression levels of PIK3R1 and AKT3 were positively correlated with the GLI1 expression level in AML patients (Fig. 3D). Using hTF target analysis (the high-quality transcription factor binding profile database), we found that the transcription factor GLI1 has potential binding sites in the PIK3R1 promoter (Fig. S4). To verify whether GLI1 binds to the PIK3R1 promoter region, we co-transfected human renal epithelial 293T cells with a PIK3R1 luciferase reporter plasmid and a GLI1 expression plasmid in reporter assays. These assays revealed that GLI1 expression triggers a twofold increase in activity in the PIK3R1 reporter (Fig. 3E). Consistent with increased reporter activity, GLI1 overexpression resulted in increased GLI1 binding to PI3K (p85) in THP-1 and U937 cells (Fig. 3F).

Because GLI1 regulates the PI3K/AKT pathway to exert its function, it is possible that inhibition of the PI3K/AKT pathway could reverse the effects of GLI1 overexpression on cell proliferation. To test this hypothesis, cell viability was determined after treatment with different concentrations of the PI3K inhibitor LY294002 or the AKT inhibitor MK-2206 2HCL in THP-1/OE and U937/OE cells. We observed that the PI3K and AKT inhibitor could reverse the effects of GLI1 overexpression on cell proliferation (Fig. 3G, H), suggesting that GLI1 promotes proliferation through the PI3K/AKT pathway. Together, these data demonstrate a PI3K-dependent mechanism in which GLI1 regulates the proliferation of AML cells.

### GLI1 significantly promotes tumor growth in a xenograft mouse model

**Fig. 4:**
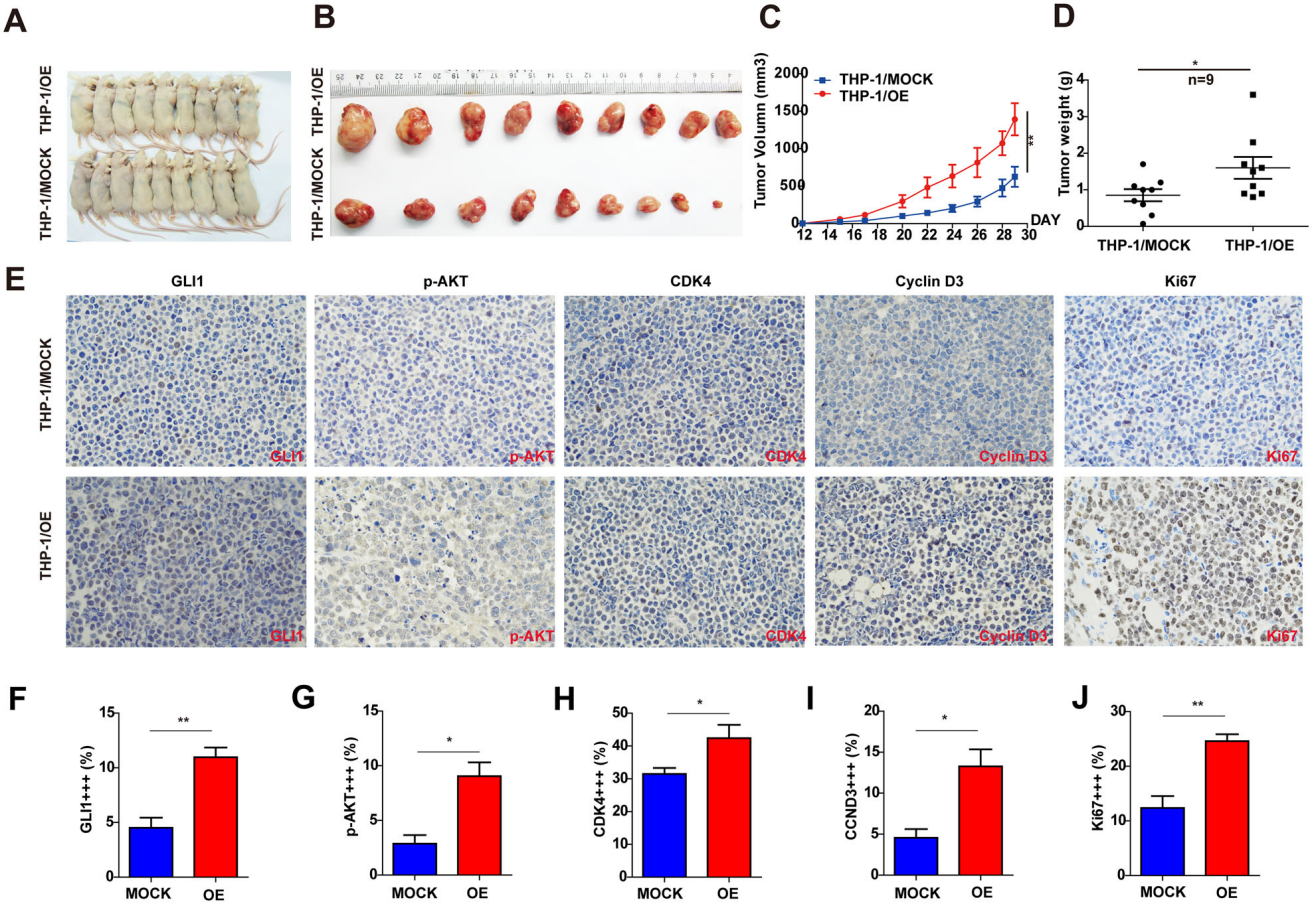
GLI1 significantly promotes tumor growth in an animal model. **A**, **B** Images of xenograft mice (**A**) and tumors (**B**) from mice injected with THP-1/OE and THP-1/MOCK cells (OE and MOCK: *n* = 9). **C**, **D** The volumes (*P* = 0.0022) and weights (*P* = 0.0370) of tumors harvested from xenograft mice injected with THP-1/OE and THP-1/MOCK cells. The *p* values for the tumor volumes were obtained by two-way ANOVA. The *p* values for the tumor weights were obtained by a two-tailed Student’s test. **E** Representative IHC staining images of the GLI1, p-AKT, cyclin D3, CDK4, and Ki67 expression levels in xenograft tumor tissues. **F**–**J** The strong positive (+++) signals from GLI1, p-AKT, Cyclin D3, CDK4, and Ki67 staining were quantified. The *p* values were obtained by a two-tailed Student’s *t*-test *P < 0.05, **P < 0.01, ***P < 0.001, ns not significant.

To test the promoting effects of GLI1 on tumor growth in vivo, we injected recipient mice with THP-1/MOCK (as a control group) or THP-1/OE (as experimental group) cells in the flanks of mice (nine mice per group), and their tumor sizes were measured every 2 days from days 12 to 29 (Fig. 4A, B). The mice were sacrificed on day 29 for ethical reasons. Although the weights of the mice were no differences between the two groups, mice injected with THP-1/OE cells suffered a heavier tumor burden as demonstrated by the larger tumor volume and increased tumor weight formed by THP-1/OE cells compared with those of tumors formed by THP-1/MOCK cells (Fig. 4C, D). To verify whether GLI1 overexpression upregulates cell cycle regulators and the PI3K/AKT pathway in this model, we examined the expression of GLI1, p-AKT, Cyclin D3, CDK4, and Ki67 in the xenograft tumor tissues using immunohistochemical staining. The staining scores for GLI1, p-AKT, CDK4, Cyclin D3, and Ki67 indicated that the expression of GLI1, p-AKT, Cyclin D3, CDK4, and Ki67 was significantly higher in the experimental group than in the control group (Fig. 4E–J). Collectively, we demonstrated that overexpression of GLI1 in AML cells promotes tumor growth and upregulates cell cycle regulators and the PI3K/AKT pathway in a xenograft model.

### Inhibition of GLI1 and CDK4/6 has a synergistic effect on promoting drug sensitivity and reducing cell viability in AML cells

**Fig. 5:**
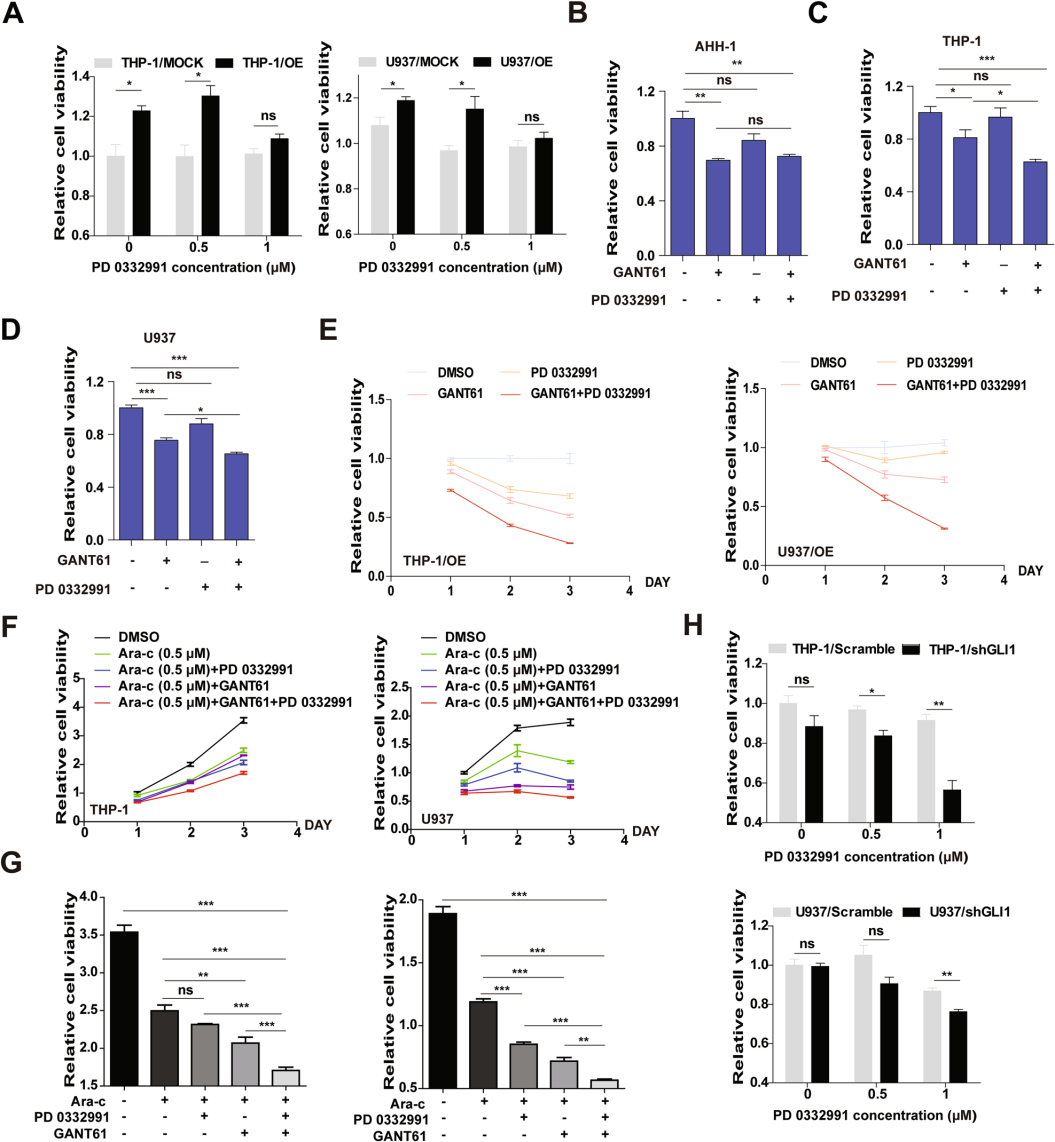
Inhibition of GLI1 and CDK4/6 had a synergistic effect on promoting drug sensitivity and reducing cell viability in AML cell lines. **A** Relative cell viability of THP-1 and U937 MOCK/ OE cells after treatment with different concentrations of PD 0332991 (0, 0.5, and 1 μM) for 48 h. **B**–**D** Relative cell viability of the normal lymphocyte cell line (AHH-1) (**B**) and the AML cell lines THP-1 (**C**) and U937 (**D**) after treatment with GANT61 and/or PD 0332991 for 24 h. **E** THP-1/OE and U937/OE cells were treated with GANT61 (20 μM) and/or PD 0332991 (0.5 μM) for 24, 48, and 72 h. Cell viability was determined by CCK8. **F** Relative cell viability of THP-1 (left) and U937 (right) cells in response to different combinations of drugs. Ara-c, PD 0332991, and GANT61 were added at concentrations of 0.5, 0.5, and 20 μM for 24, 48, and 72 h. Cell viability was determined by CCK8 at the indicated time points. **G** Statistical analysis of cell viability at 72 h in (**F**). **H** Relative cell viability of THP-1 and U937 Scramble/shGLI1 cells after treatment with different concentrations of PD 0332991 (0, 0.5, and 1 μM) for 48 h. The *P* values were obtained by a two-tailed Student’s *t*-test and two-way ANOVA. *P < 0.05, **P < 0.01, ***P < 0.001, ns not significant.

Because GLI1 overexpression regulates the proliferation and drug sensitivity of AML cells by regulating cell cycle progression and upregulating CDKs, we hypothesized that inhibition of CDKs could reduce the adverse effects of GLI1 overexpression. To test this hypothesis, AML cells (THP-1 and U937/MOCK/OE/scramble/shGLI1 cells) were treated with different concentrations of PD 0332991, a CDK4/6 inhibitor, and the viability of these treated cells was determined. Compared to the control cells, PD 0332991 could abolish the increased proliferation induced by GLI1 overexpression in both the THP-1/OE and U937/OE cell lines (Fig. 5A). AHH-1, a human peripheral blood B lymphocyte cell line was used as a negative control (Fig. S5A). Next, we assessed the effects of combined treatment of PD 0332991 and GANT61 (a GLI1 inhibitor). AHH-1, THP-1, and U937/WT/MOCK/OE cells were treated with GANT61 (20 μM) w/o PD0229331 (1 μM) for 24 h. While GANT61 led to efficient growth inhibition of THP-1/WT, U937/WT, and AHH-1 cells, PD 0332991 enhanced the GANT61-mediated cytotoxicity in THP-1/WT and U937/WT cells but not in AHH-1 cells (Fig. 5B–D). Both GANT61 and PD 0332991 reduced the viability of GLI1 OE cells, and co-treatment with GANT61 and PD 0332991 achieved the most significant inhibitory effects (Figs. 5E and S5B). Ara-c was further added to the combination to test whether its addition could result in a more significant inhibitory effect on cell viability. THP-1/WT and U937/WT cells were treated with Ara-c (0.5 μM) alone, or in combination with GANT61 (20 μM), and PD 0332991 (1 μM) for up to 72 h. Although the viability of cells treated with PD0332991 plus Ara-c was comparable to that of cells treated with Ara-c alone for THP-1 cells, GANT61 plus Ara-c and all three-drug combinations could significantly inhibit the growth of these cells. The viability in the Ara-c (0.5 μM) + GANT61 (20 μM) + PD 0332991 (1 μM) treatment group more pronouncedly decreased compared with the other treatment groups for U937 cells (Fig. 5F, G). These data revealed that the CDK4/6 inhibitor abolished the effects of GLI1 overexpression on AML cell proliferation and combined use of GANT61 and PD 0332991 enhances Ara-c drug sensitivity. We further tested whether CDK inhibition could have a synergistic effect when combined with GLI1 knockdown. As expected, GLI1 knockdown cell lines demonstrated further reduced cell viability compared with controls when treated with PD 0332991 at 1 μM for 48 h (Fig. 5H). Together, these data suggest that inhibition of GLI1 and CDK4/6 have a synergistic effect on promoting drug sensitivity and reducing cell viability in AML cells.

### Co-treatment of GANT61 and PD 0332991 were effective and safe in AML patients

**Fig. 6:**
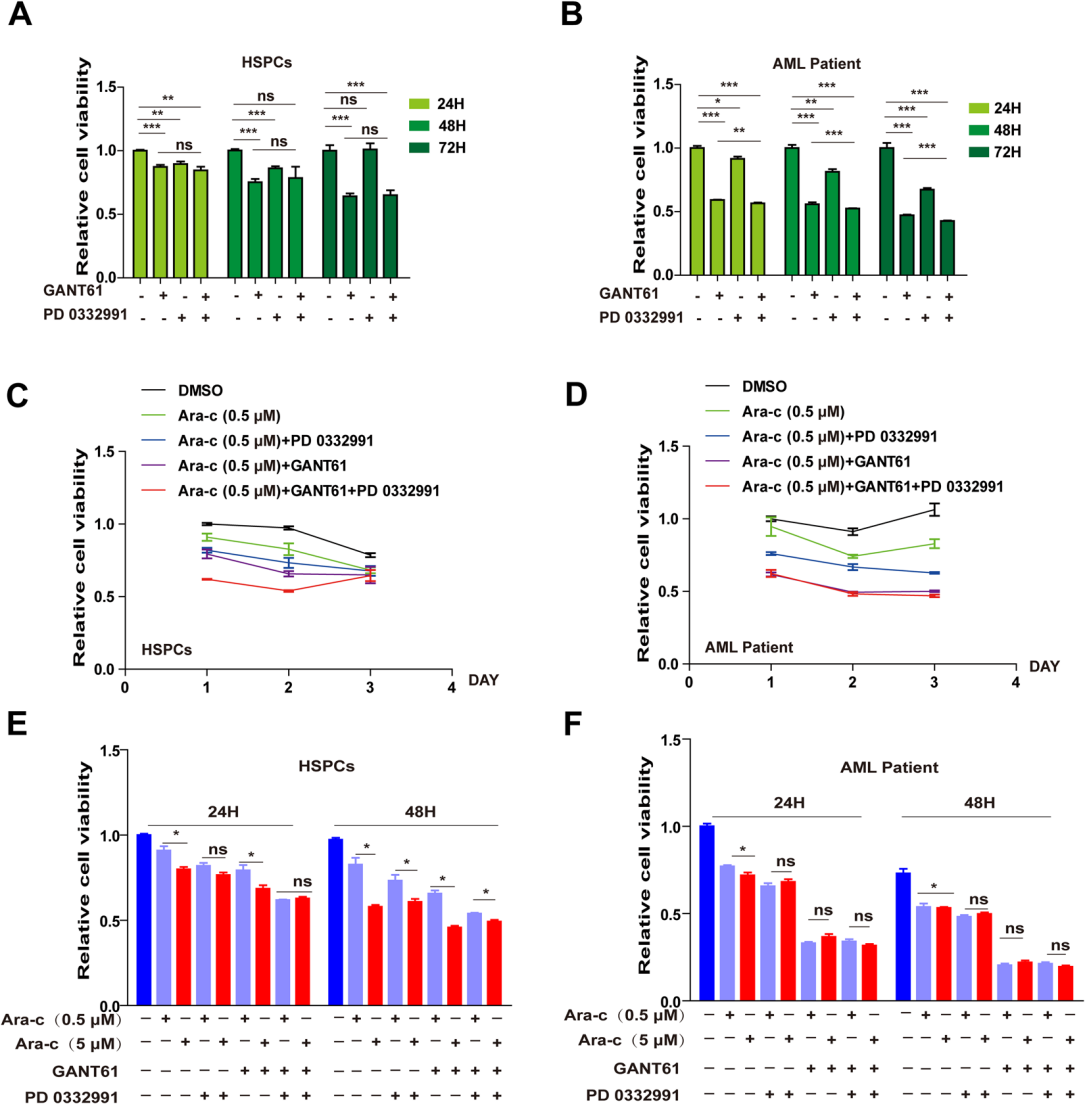
Co-treatment with GLI1 and CDK4/6 inhibitors enhanced the cytarabine sensitivity of AML patients. **A**, **B** Cell viability of HSPCs (**A**, *n* = 3) and AML patients (**B**, *n* = 3) treated with GANT61 and PD 0332991. **C**, **D** HSPCs (**C**, *n* = 3) and AML patients (**D**, *n* = 3) were treated with Ara-c (0.5 μM) and/or GANT61 (20 μM) /PD 0332991 (0.5 μM) or Ara-c (0.5 μM), GANT61 (20 μM) and PD 0332991 (0.5 μM). **E**, **F** HSPCs and AML patients (**F**) were treated with Ara-c (0.5 μM or 5 μM) and GANT61/PD 0332991 or Ara-c + GANT61 + PD 0332991 for 24 h and 48 h. Cell viability was determined by CCK8. *P* values were obtained by a two-tailed Student’s *t*-test and two-way ANOVA. *P < 0.05, **P < 0.01, ***P < 0.001, ns not significant.

We must take into account the side effects of drugs. The reduced viability of AML cells could be induced by accumulated cytotoxicity of combined drug treatment. To exclude this possibility, the cytotoxic effects of GANT61 (20 μM) and PD 0332991 (1 μM) on hematopoietic stem/progenitor cells (HSPCs) and fresh primary AML cells was assessed. No significant difference in cytotoxicity was observed between treatment with GANT61 alone and GANT61 (20 μM) + PD 0332991 (1 μM) for HSPCs although both GANT61 and PD 03033291 decreased the viability of HSPCs (Fig. 6A). In contrast, PD 0332991 could efficiently enhance the cytotoxicity of GANT61 (20 μM) in AML primary cells, which was supported by the significantly lower viability of primary AML cells treated with GANT61 (20 μM) + PD 0332991 (1 μM) (Fig. 6B). Notably, although the combination of PD 0332991 and GANT61 with Ara-c demonstrated more severe cytotoxicity for HSPCs on days 1 and 2 compared to Ara-c with either of the two, the combination of the three drugs did not lead to enhanced cytotoxicity at day 3 (Fig. 6C). In addition, combined treatment of Ara-c (0.5 μM), GANT61 (20 μM), and PD 0332991 (1 μM) demonstrated maximum inhibition of viability for primary AML cells (Fig. 6D). Notably, the cytotoxicity of a higher concentration (5 μM) of Ara-c for HSPCs was apparent (Fig. 6E). In contrast, both concentrations of Ara-c achieved similar effects when used with GANT61, PD 0332991, or both for AML primary cells (Fig. 6F). The above data suggest that PD 0332991 enhances GANT61 cytotoxicity, and PD 0332991 and GANT61 have a synergistic effect in promoting Ara-c sensitivity in GLI1-overexpressing AML cell lines and primary AML cells.

## Discussion

In this study, we report that GLI1 overexpression promotes cell proliferation and reduces the chemotherapeutic sensitivity of AML cell lines. We further demonstrate that GLI1 exerts its role through the regulation of the cell cycle. Interestingly, we found that GLI1 knockdown sensitized THP-1 and U937 cells to Ara-c but not ADR treatment (Fig. 1I, J). It is possible that ADR is efficient in terms of inhibiting proliferation; thus, the addition of GLI1 knockdown failed to demonstrate significant synergism with ADR. Instead, GLI1 knockdown synergized with Ara-c to inhibit the growth of AML cells, particularly at low doses. We found that GLI1 overexpression upregulated CCND1, and GLI1 knockdown specifically downregulated CCNB and CCND1 (Fig. S2B, C). Ara-c, a nucleoside analog, causes S phase cell cycle arrest, inhibits DNA polymerase, and directly regulates the cell cycle genes CDK4 and cyclin D1 (CCND1), subsequently leading to cell cycle arrest in the G1/S phase^32^. Therefore, the synergism between GLI1 knockdown and Ara-c is likely achieved by targeting different phases of the cell cycle.

We demonstrated that GLI1 regulates phosphorylation of PI3K and AKT but not their expression level. We also demonstrated that GLI1 could directly bind PI3KR1. However, the mechanism by which GLI1 regulates the phosphorylation of PI3K and AKT remains unknown. Moreover, we demonstrated that inhibition of the PI3K/AKT pathway could reverse the effects of GLI1 expression. Interestingly, PI3K and AKT inhibitors did not inhibit the expression of GLI1^30^. These data suggest that GLI1 is the upstream regulator of the GLI1-PI3K/AKT axis. Further study is needed to illustrate the mechanism by which GLI1 alters the phosphorylation of PI3K and AKT.

**Fig. 7:**
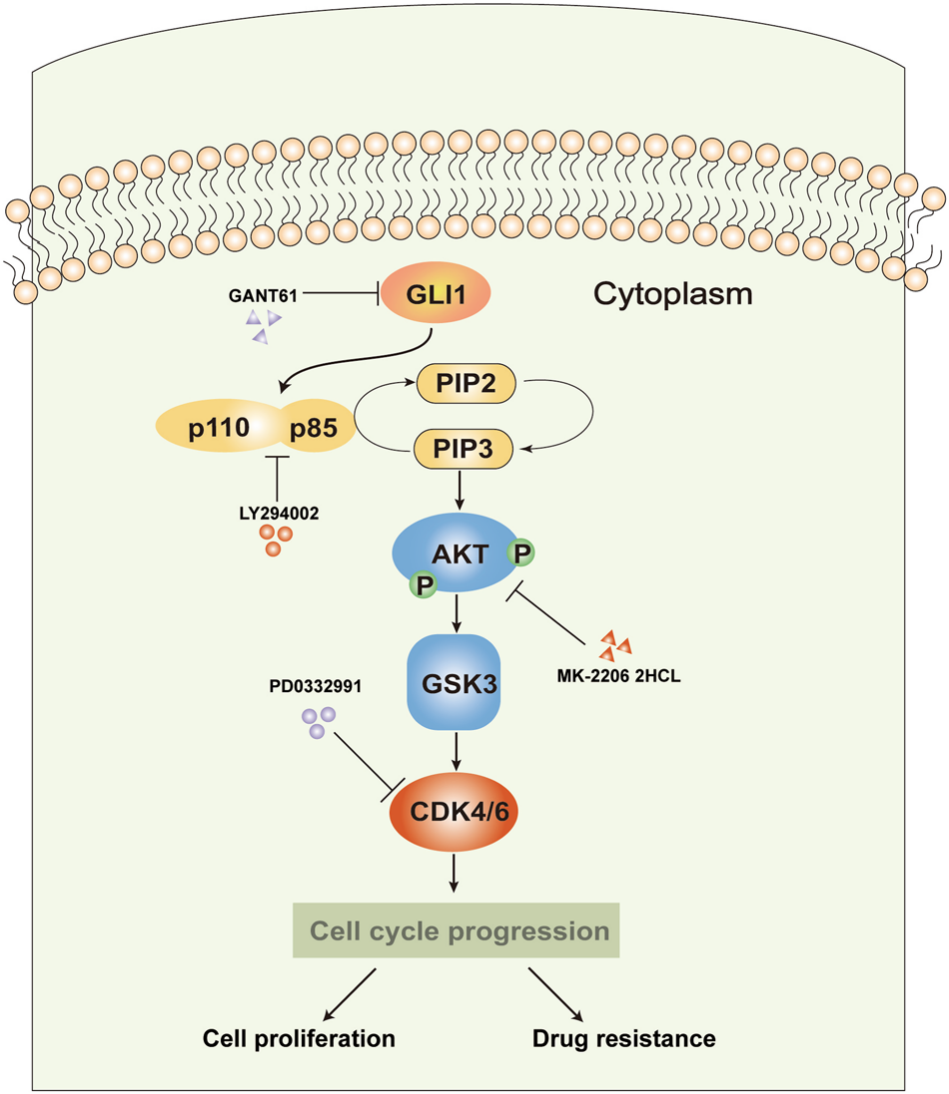
Schematic drawing of the mechanism by which GLI1 regulates cell proliferation and drug sensitivity in AML. GLI1, a representative transcription factor in the Hedgehog (Hh) signaling pathway, directly activates PI3K/AKT pathway and subsequently up-regulates CDK4/6 protein through regulating GSK3.

We further reported that inhibition of GLI1 and CDKs had a synergistic effect on promoting drug sensitivity in AML cells. The combination of the GLI inhibitor GANT61 and the CDK4/6 inhibitor PD 0332991 with Ara-c demonstrated significant synergistic effects in THP-1 cells. In addition, we found that PI3K, AKT, and CDK4/6 inhibitors could reverse the promoting effects of GLI1 overexpression on cell proliferation, while the AKT inhibitor could reverse the increase in GSK3α/β and CDK by GLI1 overexpression. Collectively, our data suggest that GLI1 functions by activating the PI3K/AKT/GSK3β/CDK signaling pathway, leading to cell growth and drug resistance in AML cells (Fig. 7). This finding provides a rationale for combinatory therapy including the simultaneous inhibition of GLI1 and CDKs to treat AML.

Crosstalk between two pathways is a leading cause of drug resistance in cancer. After an initial response to treatment, resistance quickly develops due to reciprocal activation of the target molecule, including by upstream or negative feedback mechanisms by down-stream molecules^33,34^. Our study demonstrated that GLI1 was an upstream effector of PI3K. Recent evidence has outlined the importance of GLI1 and PI3K functioning independently of one another in cancer. However, co-inhibition of GLI1 and PI3K had apparently enhanced toxic effects in HSPCs, which raised serious concerns about the side effects of this treatment^30^. Therefore, to reduce the cytotoxic side effects of this co-inhibition, we attempted to target downstream CDKs instead of the PI3K/AKT pathway. Here, we demonstrate that combinatory treatment consisting of the GLI1 inhibitor GANT61 and the CDK inhibitor PD 0332991 was effective in cell lines and fresh primary patient cells, providing a novel option for treating AML in the near future. Specifically, PD 0332991 could synergize with GANT61 to efficiently inhibit the growth of AML cells without severe cytotoxicity in AHH-1 cells. The advantage of the combination of PD 0332991 and GANT61 was also demonstrated in AML patient samples and normal HSPCs. Further validation of the effectiveness of GANT61 and PD 0332991 in treating CDX and PDX mouse models is needed in future studies. Additionally, several studies have suggested that combination treatment is superior to treatment with a single targeted agent^35,36^. Another benefit of combination treatment is that the dose of each drug can be decreased, which further reduces the risk of toxicity to normal cells.

In summary, GLI1 and p-AKT expression were closely associated with AML. GLI1 activated the PI3K/AKT pathway, and co-expression of GLI1 and p-AKT was associated with cell proliferation, drug-resistance, and an increase in the percentage of cells in the S + G2 phase, indicating poor prognosis. Targeted agents that act against both GLI1 and CDK4/6 may overcome drug resistance and achieve better inhibitory effects and enhanced Ara-c chemo-sensitivity. This study provides a new perspective for using GLI1 and CDK4/6 inhibitors to treat relapsed/refractory (RR) patients.

## Materials and methods

### Patients samples

Liquid bone marrow samples were collected from three AML-RR patients who were diagnosed with AML according to 2008 WHO criteria and treated at Xiangya Hospital of Central South University, Hunan, China. For comparison, three healthy volunteer donors were included as controls. The experimental protocols were approved by the ethical committee of Xiangya Hospital, Central South University. Informed consent was obtained from all research subjects.

### Cell culture

THP-1, U937, and 293T cells were obtained from the Cell Resource Center (Xiangya Medical College, Central South University, Hunan, China). the 293T cells were cultured in a DMEM medium (Corning, Inc). The other cells were maintained in RPMI-1640 medium (Corning, Inc). All media was supplemented with 10% fetal bovine serum (Corning, Inc.) and 1% antibiotic solution including penicillin/streptomycin (Sigma, MO, USA). All cells were incubated at 37 °C in a humidified atmosphere of 5% CO_2_.

### Lentiviral transduction of cell lines

THP-1 and U937 cells were resuspended in enhanced infection solution. A total of 5 × 10^4^ cells/mL per cell line was seeded into 96-well plates (three replicates for each cell line). Mock infection, GLI1 overexpression (GLI1/OE), GLI1 scramble, and GLI1 shGLI1 lentiviruses (Shanghai Genechem) at a titer of 1 × 10^8^ TU/ml were added to the corresponding wells for transduction (MOI: 20-50), and polybrene was added at 1:1000. After incubation in 5% CO_2_ and 37 °C in an incubator for 12 h, successful transduction of the intended cells was confirmed with a fluorescence microscope. Stably transduced OE and knockdown cells were selected with puromycin at a concentration of 2 µg/ml.

### Western blot analysis

Equal amounts of protein were solubilized in sample buffer and electrophoresed in denaturing 10% SDS-polyacrylamide gels and then transferred to polyvinylidene fluoride (PVDF) membranes (Millipore, Billerica, MA, USA). The membranes were saturated in TBST containing 5% BSA (Bio Sharp Sigma, A-4612) for an hour at room temperature and incubated with primary antibodies overnight at 4 °C. After incubation with a secondary antibody, the blots were then washed and detected with the ChemiDoc MP System (Bio-Rad Laboratories. Inc., Hercules, CA, USA). The antibodies used are as follows: GLI1 (Cell Signaling, 3538), p-AKT (Ser473) (Cell Signaling, 4060), AKT (Cell Signaling, 9272), p-PI3K (Cell Signaling, 17366), PI3K (Cell Signaling, 4257), GSK3α/β (Bioworld, BS1412), Cyclin D1 (Cell Signaling, 2978), CyclinD2 (Proteintech,10937-1-AP), CyclinD3 (Proteintech, 26755-1-AP), CDK4 (Proteintech, 11026-AP), CDK6 (Proteintech,19117-1-AP), GAPDH (Santa Cruz, CA, USA), and anti-rabbit IgG (Proteintech, SA00001-2).

### Quantitative RT-PCR

RNA was isolated from 1 × 10^6^ cells using TRIzol reagent (TaKaRa, Japan, Cat#9109). Total RNA (1000 ng) was reverse transcribed into first-strand cDNA using the Prime Script^TM^ RT Reagent Kit (TaKaRa, Japan, Cat#RR047A). cDNA was amplified in a total volume of 20 μl using a transcription kit (TaKaRa, Japan, Cat#RR047A). Reactions were run on an Applied Biosystems Prism machine using ABI StepOnePlus (Applied Biosystems, Foster City, CA, USA). The thermal cycler conditions were as follows: 95 °C for 30 s followed by 40 cycles of 95 °C for 5 s, 60 °C for 34 s, and 95 °C for 15 s, 60 °C for 1 min, and 95 °C for 15 s.

### Cell viability assay

Cell viability was determined by a Cell Counting Kit-8 assay (7sea biotech, China) after treatment. THP-1 and U937 cells (5 × 10^4^/ml) were seeded in 96-well culture plates without drug treatment or incubated with GANT61 (Adooq Bioscience, A13252), LY294002 (Adooq Bioscience, A10547), MK-2206 2HCL (Topscience, T1952), PD 0332991 (La Jolla, CA), ADR (Medcheme Xpress, HY-15142), and Ara-c (Solarbio, Lot. No. 317B002). After culture for the indicated time, 10 μL CCK8 solution was added to each well for a 3 h culture at 37 °C. Absorbance was measured by a spectrophotometer (Bio Tek Instruments, US) at a wavelength of 450 nm. The cell viability rate = (1—OD value of treatment/OD value of control).

### Cell cycle analysis

The cell cycle was analyzed using the Cell Cycle Staining Kit (Liankebio, China). A total of 1 × 10^6^ cells were seeded in 12-well plates for 24 h. The cells were harvested, washed twice with 1× PBS, and incubated with 1 μl DNA staining solution and 10 μl permeabilization solution (PI) in the dark for 30 min at room temperature. The cell cycle was analyzed using a flow cytometer (Becton Dickinson, CA, USA).

### Detection and quantification of PI(3,4,5)P3

The PI(3,4,5)P3 (PIP3) level in AML cell lines (THP-1, U937) with GLI1 overexpression was assessed using the PIP3 Mass ELISA Kit (Shanghai Kexing Co., Ltd, F9722-A) following the manufacturer’s instructions. The PIP3 mass was normalized by the cell number of each sample.

### Coimmunoprecipitation

An appropriate 1 ml of lysate was added to the cell culture plate, which was placed on ice for 2 h for full lysis. The lysates were centrifuged at 4 °C, 14,000 g for 15 min, and the supernatant was collected. A small amount of lysate was used for subsequent WB analysis. The appropriate antibody (1 mg) was added to the remaining lysate, which was gently shaken and incubated overnight at 4 °C. Protein A agarose beads were washed three times with an appropriate amount of lysis buffer and centrifuged at 4 °C, 1000 g for 10 min. Pretreated protein A agarose beads were added to the cell lysate, incubated for 4 h at 4 °C with gentle shaking and centrifugation at 4 °C, 1000 g for 10 min. The supernatant was removed with a pipette, and the agarose beads were washed three times with 1 mL of lysis buffer and centrifuged at 4 °C,14,000 g for 5 s. Then, 40–80 μl of 1× SDS loading buffer was added, and samples were incubated in a 100 °C metal bath for 5 mins and subjected to WB analysis.

### Luciferase assays

The 293T cells seeded in 96-well plates (2 × 10^4^ cells per well) were transfected with 100 ng luciferase reporter plasmid, 100 ng expression plasmid, and 20 ng of the constitutive reporter plasmid pRL-TK for luciferase activity normalization. An empty vector was added when needed so that all transfection reactions contained a total of 250 ng DNA. Forty-eight hours after transfection, cell lysis and enzymatic activity analysis were performed using the Dual-Glo Luciferase Assay system (Promega, E2920) following the manufacturer’s instructions. Results are presented as the means of three biologically independent duplicates.

### Xenograft model

Nude mice (nu/nu, female 4–6 weeks old) were randomly allocated to the MOCK and OE group (*n* = 9 for each group) and then subcutaneously injected with 2 × 10^6^ THP-1 MOCK/OE cells in the flank. Tumor sizes were measured every two days using a vernier caliper. Tumor growth was recorded by measurement of three perpendicular diameters using the formula (min^2^ × max)/2. The masses of the tumors (g) derived from the treatments were analyzed. The mice were sacrificed, and the tumors were harvested and weighed on day 29. Measurement and characterization of xenograft models were done with an awareness of group allocation.

### Immunohistochemical staining

GLI1, p-AKT, cyclin D3, CDK4, and Ki67 staining were performed. Briefly, resected tumors from xenograft mice were fixed in 10% buffered formalin, embedded in paraffin, and mounted on slides. After deparaffinization and rehydration, mouse tumor sections were incubated in 3% hydrogen peroxide to suppress endogenous peroxidase activity. Antigen retrieval was achieved by microwaving the sections in 10 mM sodium citrate (pH 6.0). To block the sections, 10% goat serum was then used. Human GLI1, p-AKT, cyclin D3, CDK4, and Ki67 (Abcam) antibodies were incubated with the mouse tumor sections overnight at 4 °C. Detection was performed with the Dako IHC kit (Dako EnVision+ System, HRP, Agilent technologies). Slides were stained with 3,30-diaminobenzidine (Sigma-Aldrich), washed, counterstained with hematoxylin (Sigma-Aldrich), dehydrated, and mounted. Images of each slide were taken using an inverted microscope for data analysis.

### Statistical analysis

In this study, differences among these groups were examined by Student’s *t*-test or two-way ANOVA as appropriate. A *P* value < 0.05 was considered statistically significant. Data are represented as the means ± SD of vehicle controls. Graphs were drawn using GraphPad Prism 5 software.

## Supplementary information

Author contribution form

Reproducibility Checklist form

Supplementary figure S1

Supplementary figure S2

Supplementary figure S3

Supplementary figure S4

Supplementary figure S5

Supplementary methods
