# Supplementary material for: GLI1 reduces drug sensitivity by regulating cell cycle through PI3K/AKT/GSK3/CDK pathway in acute myeloid leukemia
Source: Cell Death Dis. 2021 Mar 3;12(3):231. doi: 10.1038/s41419-021-03504-2 (PMC7930050; doi:10.1038/s41419-021-03504-2)
Supplement: Supplementary file 1 — Author contribution form [file 41419_2021_3504_MOESM1_ESM.pdf]

DECLARATION OF CONTRIBUTIONS TO ARTICLE

ADMC

Manuscript Number:	Journal Name:	
CCDIS-20-3973	*Cell Death & Disease*	(the 'Journal')

Proposed Title of the Contribution:

GLI1 reduces drug sensitivity by regulating cell cycle through PI3K/AKT/GSK3/CDK pathway in acute myeloid leukemia (the 'Contribution)

Author(s):

Cheng zhou, Juan Du, Liang Zhao, Wei Liu, Tianming Zhao, Hui Liang, Peng Fang, Kaixuan Zhang, Hui Zeng (the 'Authors')

For all *CDDis* articles, each person named as an author in the published version must be able to show he or she has contributed substantially to the article.

Authorship credit should be based on 1) substantial contributions to conception and design, acquisition of data, or analysis and interpretation of data; 2) drafting the article or revising it critically for important intellectual content; and 3) final approval of the version to be published. Authors should meet conditions 1, 2 and 3.

Any person who cannot be shown to have made a substantial contribution to the article cannot be listed as an author in the final version. The name of any person who is deemed to have made a minor contribution can, however, appear in the Acknowledgments section of the article.

Please complete the table below to indicate the contributions of all named authors to the manuscript.

Author Full Name:	Specification of Contribution to the Manuscript:
Cheng Zhou	performed experiments, analyzed the data, wrote the manuscript
Juan Du	wrote the manuscript
Liang Zhao	experimental technical assiatance
Wei Liu	experimental technical assistance
Tianming Zhao	drew schematic figure
Hui Liang	collected clinical samples
Peng Fang	collected clinical samples
Kaixuan Zhang	collected clinical samples
Hui Zeng	designed the project, analyzed the data, wrote the manuscript, appvoed the final version

Please complete the table below to indicate the contributions of all named authors to the figures.

Figure 1:

Cheng Zhou, Juan Du

Figure 2:

Chengzhou, Wei Liu, Hui Liang

Figure 3:

Cheng Zhou, Liang Zhao

Figure 4:

Cheng Zhou, Juan Du

Figure 5:

Cheng Zhou, Hui Liang, Peng Fang, Kaixuan Zhang

Figure 6:

Cheng Zhou, Juan Du
Figure7:
Tianming Zhao, Juan Du, Cheng Zhou

Signed for and on behalf of the Author(s):	Print Name:	Date:
Hui Zeng	Hui Zeng	19 Oct 2020
