# Supplementary figures and images for: GLI1 reduces drug sensitivity by regulating cell cycle through PI3K/AKT/GSK3/CDK pathway in acute myeloid leukemia
Source: Cell Death Dis. 2021 Mar 3;12(3):231. doi: 10.1038/s41419-021-03504-2 (PMC7930050; doi:10.1038/s41419-021-03504-2)

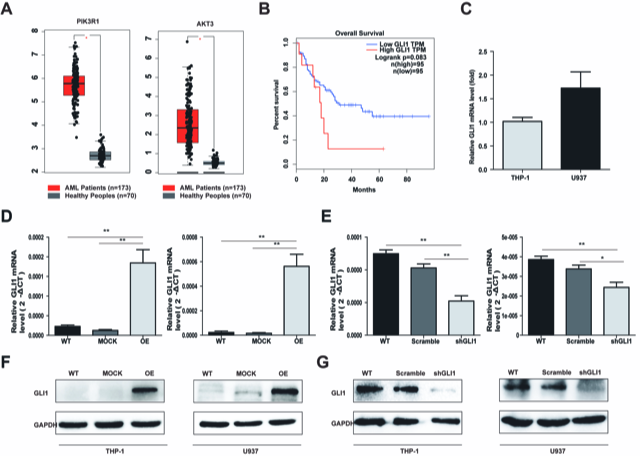

Supplement: Supplementary file 3 — Supplementary figure S1 [file 41419_2021_3504_MOESM3_ESM.tiff]

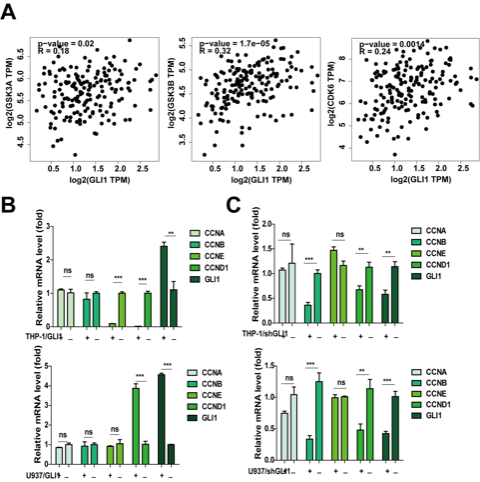

Supplement: Supplementary file 4 — Supplementary figure S2 [file 41419_2021_3504_MOESM4_ESM.tiff]

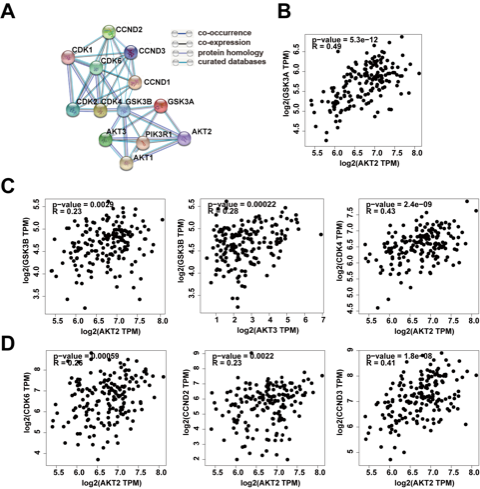

Supplement: Supplementary file 5 — Supplementary figure S3 [file 41419_2021_3504_MOESM5_ESM.tiff]

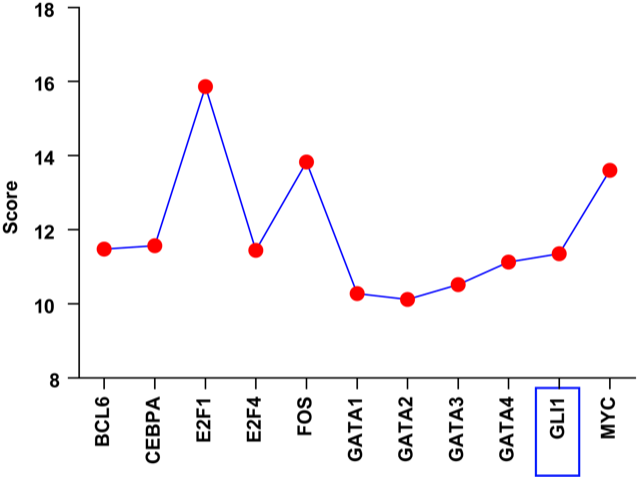

Supplement: Supplementary file 6 — Supplementary figure S4 [file 41419_2021_3504_MOESM6_ESM.tiff]

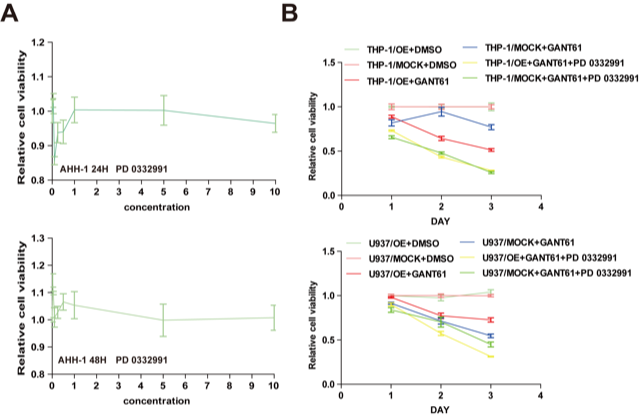

Supplement: Supplementary file 7 — Supplementary figure S5 [file 41419_2021_3504_MOESM7_ESM.tiff]
