# Supplementary material for: GLI1 reduces drug sensitivity by regulating cell cycle through PI3K/AKT/GSK3/CDK pathway in acute myeloid leukemia
Source: Cell Death Dis. 2021 Mar 3;12(3):231. doi: 10.1038/s41419-021-03504-2 (PMC7930050; doi:10.1038/s41419-021-03504-2)
Supplement: Supplementary file 8 — Supplementary methods [file 41419_2021_3504_MOESM8_ESM.docx]

**Supplementary methods**

**Primers**

Amplification primers: GLI1: forward: 5’-AACGCTATACAGATCCTAGCTCG-3’ and reverse: 5’-GTGCCGTTTGGTCACATGG-3’; GAPDH: forward: 5’-CTTTGTCAAGCTCATTTCCTGG-3’ and reverse: 5’-TCTTCCTCTTGTGCTCTTGC-3’; CCNA: forward: 5’-CGCTGGCGGTACTGAAGTC-3’ and reverse: 5’-GAGGAACGGTGACATGCTCAT-3’; CCNB: forward: 5’-TCGCATCAAACTCTCTGGCTA-3’ and reverse: 5’-TGAGCGACTAAACTCACCACT-3’; CCNE: forward: 5’-AAGGAGCGGGACACCATGA-3’ and reverse: 5’-ACGGTCACGTTTGCCTTCC-3’; CCND1: forward: 5’-GCTGCGAAGTGGAAACCATC-3’ and reverse: 5’-GCTGCGAAGTGGAAACCATC-3’.

**Supplementary figures and figure legends**

**
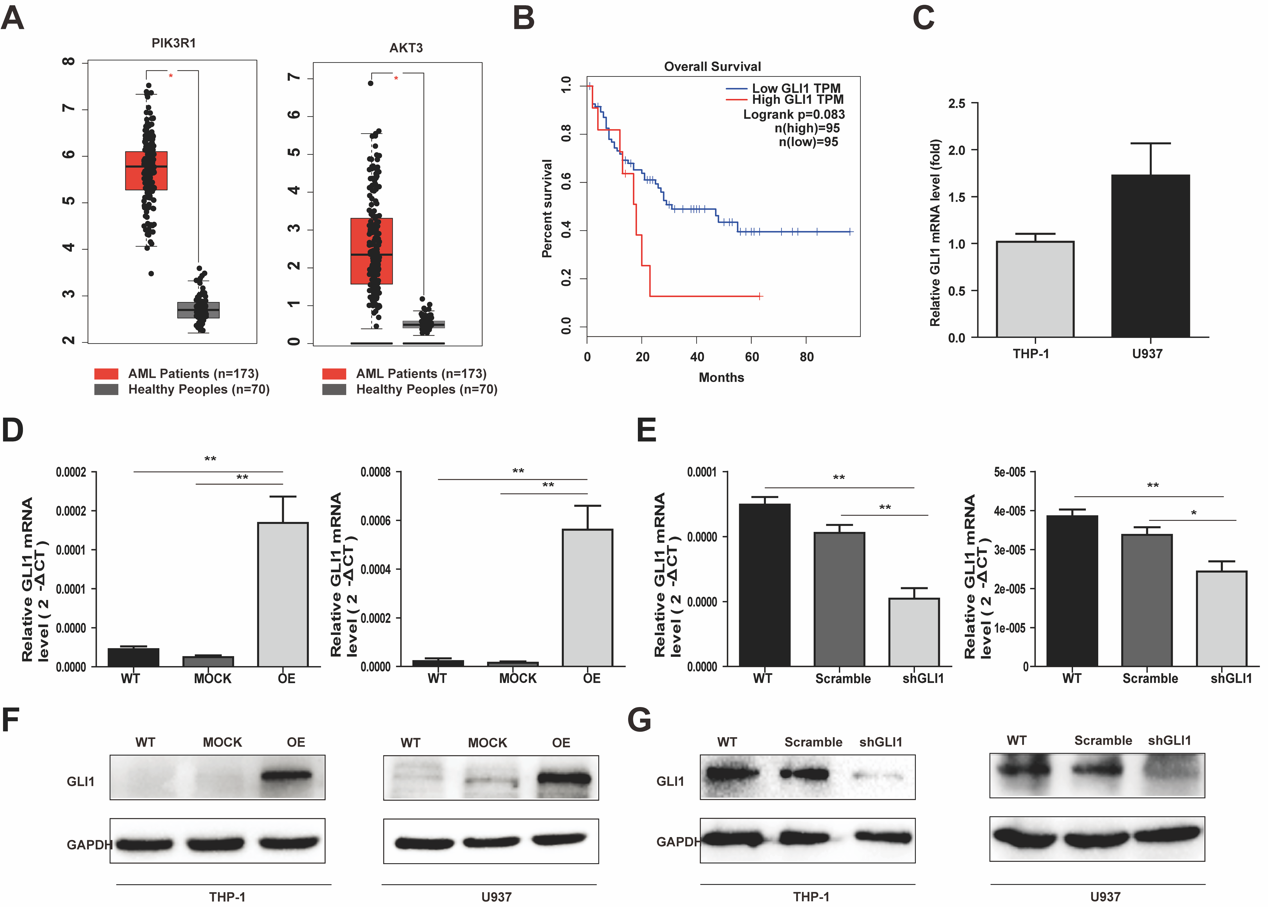
**

**Figure S1**. **The expression of PIK3R1 and AKT3 was significant higher in AML patients than in healthy people.** (A) PIK3R1 and AKT3 gene expression in AML patients and healthy people using log_2_ (TPM+1) for a log scale. (B) Kaplan-Meier curve of GLI1 expression in AML patients from the GEPIA database. (C) Endogenous GLI1 mRNA expression level in THP-1 and U937 cell lines. (D-E) GLI1 mRNA expression levels in THP-1/OE and U937/OE (D) and THP-1/shGLI1 and U937/shGLI1 (E) cells. (F-G) Protein expression levels of GLI1 in (F) and (G). WT, wild-type cell lines without manipulation. P values were obtained by two-way ANOVA.*, P <0.05, **, P <0.01, ***, P <0.001, ns, not significant.


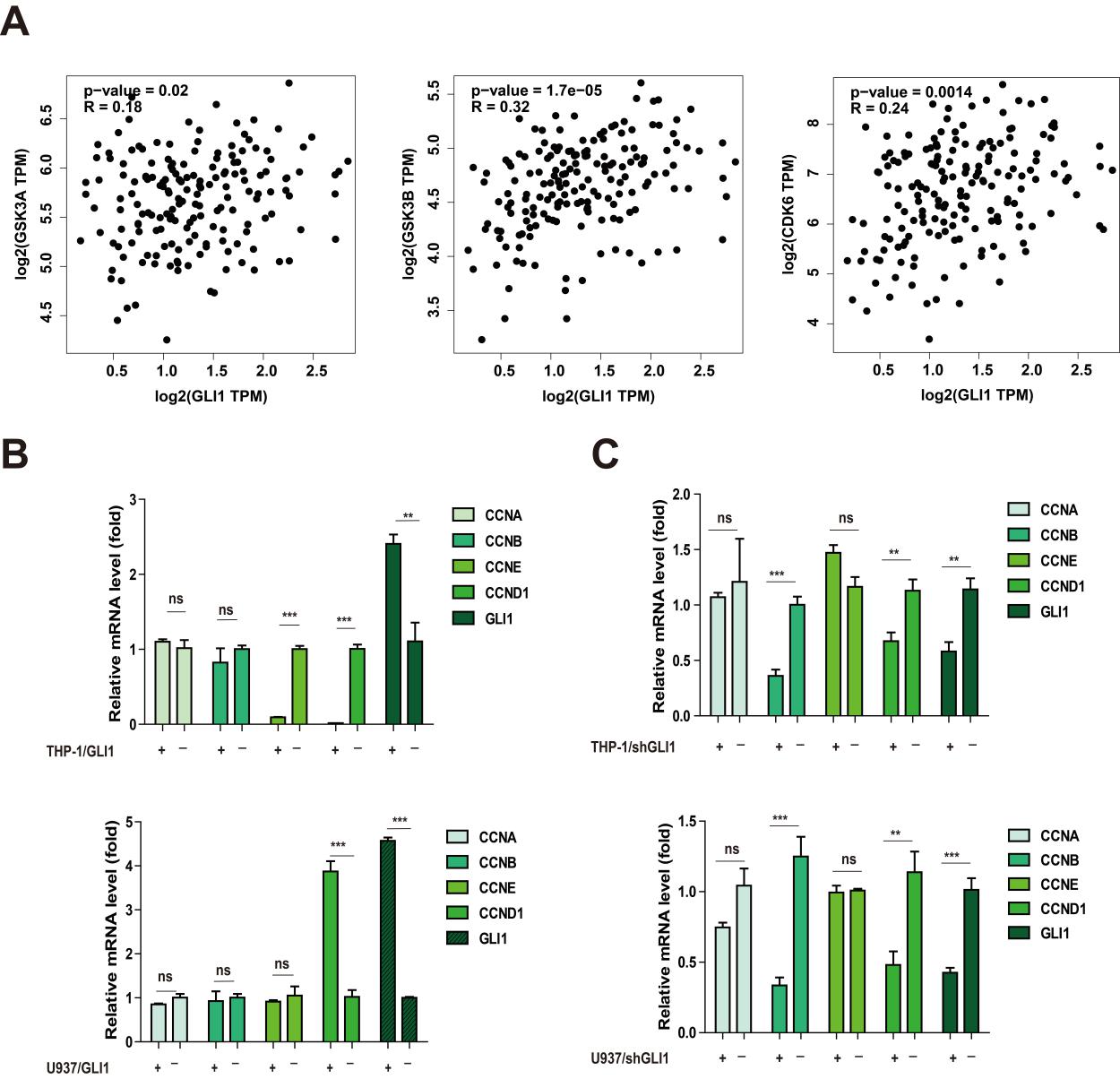


**Figure S2. GLI1 regulates CCND1 transcription in AML cell lines.** (A) Correlations between GLI1 and GSK3A/B, GLI1, and CDK6 in AML patients were evaluated using a non-log scale for calculation and a log-scale axis for visualization. The coefficient of correlation (r) and the p value are indicated. (B, C) The mRNA expression levels of cell cycle-related genes in THP-1 and U937 MOCK/OE/shGLI1/Scramble cells by RT-PCR. P values were obtained by a two-tailed Student’s t-test and two-way ANOVA. *, P <0.05, **, P <0.01, ***, P <0.001, ns, not significant.


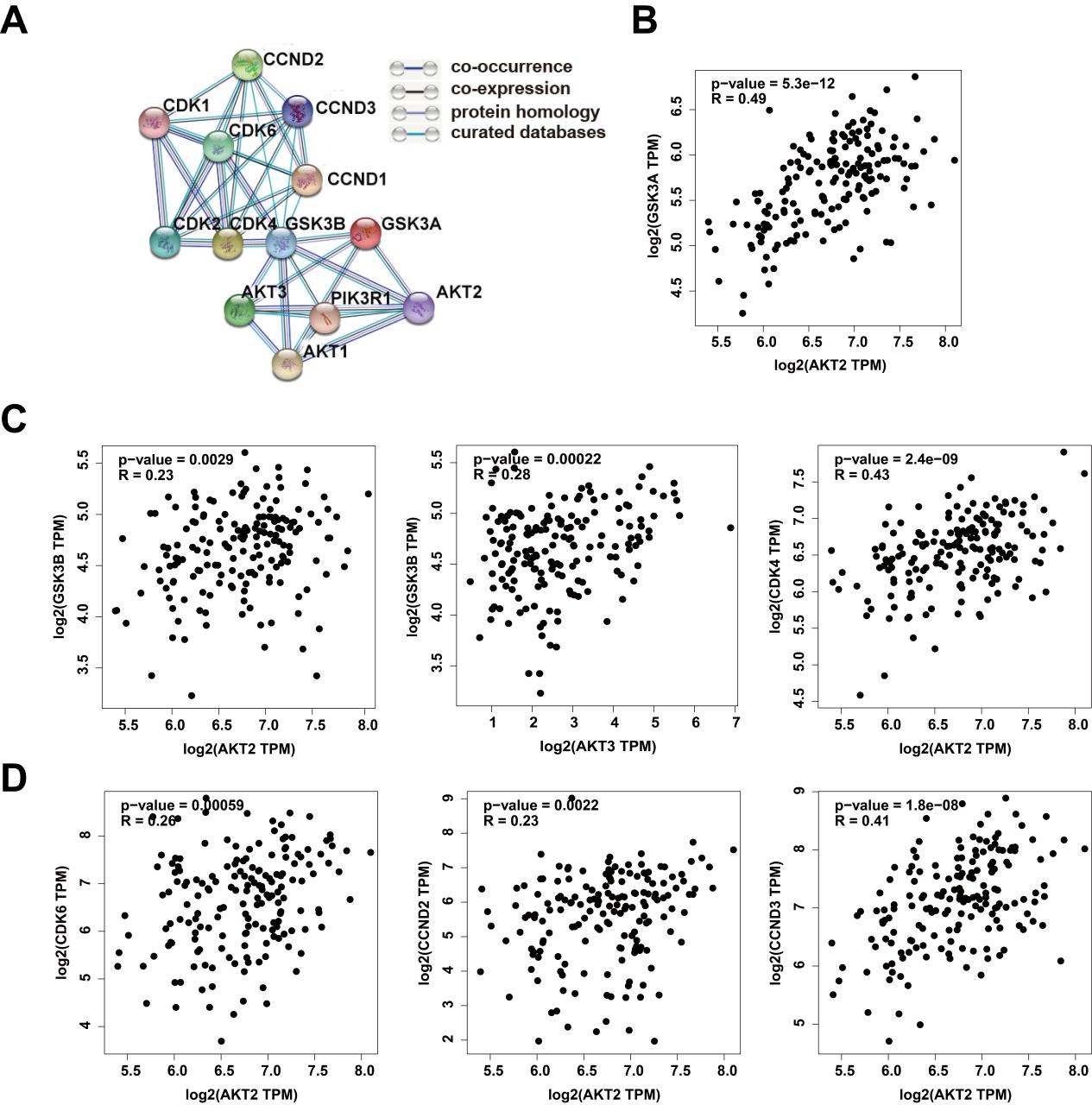


**Figure S3. AKT was positively correlated with GSK3A/B, CDKs, and CCNDs.** (A) Protein-protein interaction network of 13 target genes from the STRING database. (B-D) Correlation of AKT and GSK3A/B, AKT and cyclin Ds, AKT, and CDKs in AML patients.


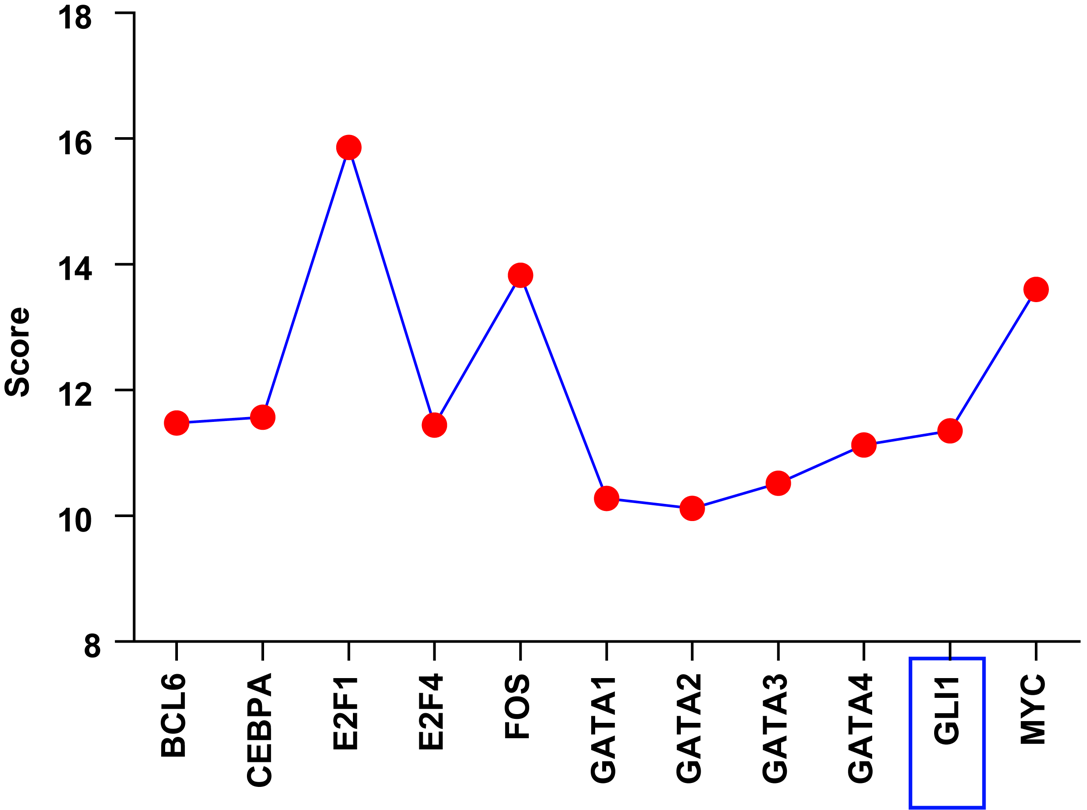


**Figure S4. Predicted binding sites for transcription factors in the PIK3R1 promoter using hTF.**


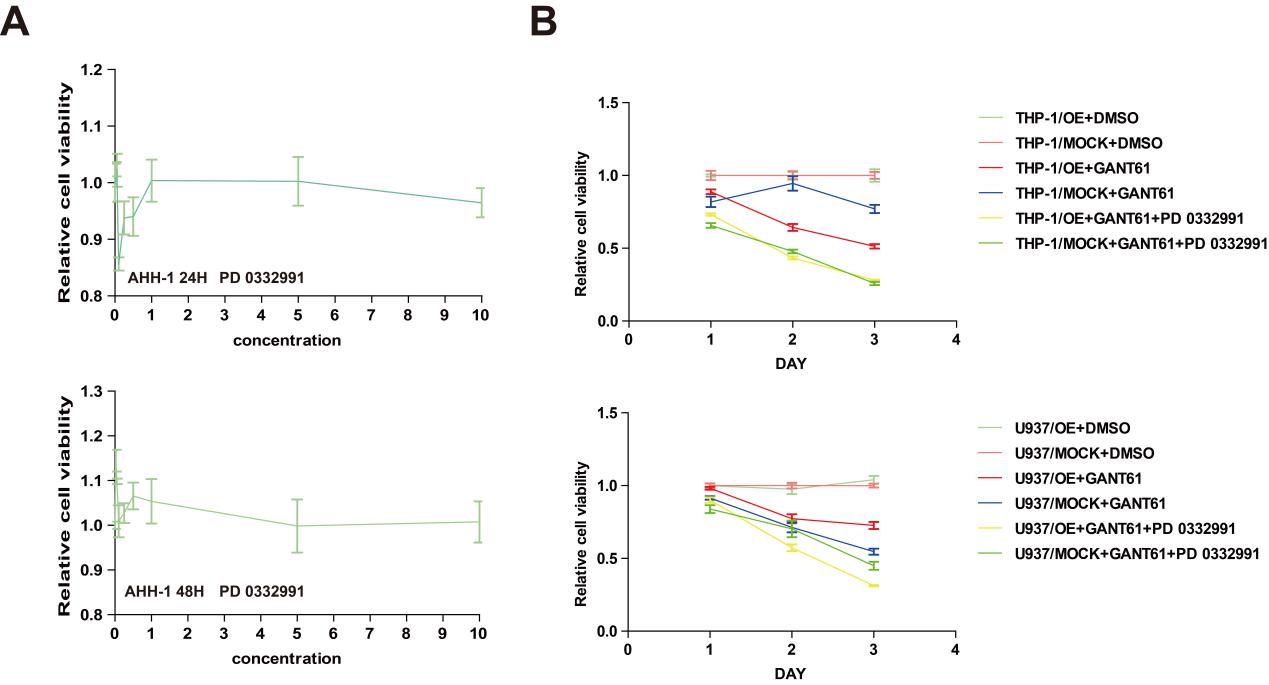


**Figure S5. PD 0332991 enhances GANT61 cytotoxicity in AML cell lines.** (A) The viability of normal lymphocytes (AHH-1) treated with different concentrations of PD 0332991 for 24 h (upper panel) and 48 h (lower panel). (B) The viability of THP-1/OE/MOCK and U937/OE/MOCK cells when treated with GANT61 (20 μM) w/o PD0332991 (0.5 μM). P values were obtained by a two-tailed Student’s t-test and two-way ANOVA . *, P <0.05, **, P <0.01, ***, P <0.001, ns, not significant.
